# Difficulties of Pressure Anesthesia

**Published:** 1905-04-15

**Authors:** J. B. Pherrin


### DIFFICULTIES OF PRESSURE ANESTHESIA.

BY J. B. PHERRIN, D.D.S.

This valuable method of producing insensibility of the tooth pulp, and which is to a considerable extent employed for the desensitization of the dentinal fibrils, much to our annoyance is attended by some hindrances which it is the purpose of this paper to discuss. In order that I may not be misunderstood, I will state that it is not for the purpose of condemning the procedure, but rather to give some helpful hints, if possible, that the process may be of still greater help to those who have met with disappointments. The method, I take for granted, is sufficiently familiar or has been described by the other papers read before you relative to this subject.

The first serious obstacle in the use of this method is the large amount of cocain sometimes used and the idiosyncrasy that is sometimes manifested by some patients. A large open apex may permit the too sudden diffusion of a large portion of the drug, and the usual symptoms of cocain are the result. It has been my great misfortune to have at least three serious cases of this sort. The result has been that I now use a much smaller portion of the cocain and wait a very little longer for results. The pulp-canal is the most perfect of all hypodermic syringes, and we must not place any diffusible drug therein that we would not wish to introduce into the general circulation.

In direct contrast to the hindrance just mentioned, two cases of immunity have been encountered, in which not the slightest effect of the drug was appreciable. The pulps were finally treated and removed after the arsenic method. The pulps were found to be normal in every respect so far as could be determined by common methods of examination. No clot congestion, pulp nodules, and but slight inflammation was found.

The exposure in each case was complete, and yet the last application of pressure was seemingly as painful as the first. In each case a little more than one-half grain of cocain was used, with no characteristic symptoms of the drug being evidenced. A few other cases have at first seemed to be immune, which, after patient effort, yielded to the inevitable.

Alcohol or a weak solution of formalins seem to aid in the absorption of the cocain-adrenalin solution, yet one case yielded readily to the solution after treatment with phenol which had stubbornly resisted the solution and the ordinary adjuvants. I can give no scientific reason for the treatment by phenol, as it is a coagulant of albuminoids and is at least theoretically contra-indicated.

Clot is another serious hindrance to the proper desensitization of the pulp. Any decay of the leathery variety is a barrier to the anesthetic, and must be removed before the operation can be successfully performed.

A secondary deposit of dentin is probably the most frequently encountered, and is often the source of much annoyance. To overcome this difficulty a particularly sensitive spot should be selected in the cavity, usually near the enamel, as sensitive dentin indicates dentinal tubuli, and usually, after one or two attempts, the operation may be performed painlessly.

Congestion should be mentioned as a really troublesome condition, the philosophy of which is easily understood. Sometimes we find a tooth extremely sensitive to the process of cutting and mistake it for normal dentin, while in reality it is dead tooth bone, and upon opening the pulp-chamber we find above the living pulp a quantity of pus, which of course prevented the action of the drug upon the living tissue. As a side note for your consideration, I will state that I have experienced considerable after-trouble in such cases from dental abscess. The theory as to the probable cause is that infection was carried to the apical region and later manifested itself in the form of an alveolar abscess.

I have noticed that a large apical foramen is often the cause of unsatisfactory results because of the too-rapid absorption of the minute quantity of the drug used, while in exceedingly small canals the pulp was more readily desensitized.

Accessibility and the form of the cavity enter largely as factors tending toward an easy operation or a difficult one. A number of cases having interesting peculiarities might be mentioned.

I am convinced that the dangerous symptoms noted were the result of the too rapid exhibition of the drug and in larger quantity than necessary. I am convinced also that one forty-eighth of a grain is sufficient for the ordinary case if time is afforded the drug to produce the physiological effect.

Sensitive dentin perhaps more than any other one thing has annoyed the dental profession. With care and patience sorrow flees from the office of the man who applies this method. The mode of exhibition of the drug is precisely the same for desensitization as for pulp extirpation. The hemorrhage resulting from immediate pulp extirpation is nicely controlled by a second or third application of adrenalin solution without the cocain.

In all the operations made in my office the adrenalin-chlorid solution, 1 to 1,000, and cocain tablet No. 150 are used. Both are prepared by Parke, Davis & Co. Each tablet contains one-sixth of a grain of cocain. The tablets are particularly superior to other forms because of their size, form, and rapid solubility.

I had hoped to give a glowing report upon the use of chloretone and suprenal liquid, but up to the present time it has been found to be less rapid in diffusibility, although a few cases might be cited where pulps have been removed painlessly by its use without the aid of cocain.

